# Transient Hyperglycemia in a Patient With Type 2 Diabetes After COVID-19 Messenger RNA Vaccination: A Case Report

**DOI:** 10.7759/cureus.63983

**Published:** 2024-07-06

**Authors:** Aniebietabasi Okon-Umoren, Sean Yaphe, Andrea Smith, Karla D Passalacqua, Katarzyna Budzynska

**Affiliations:** 1 Family Medicine, Henry Ford Health System, Detroit, USA; 2 Medical Education, Henry Ford Health System, Detroit, USA

**Keywords:** type 2 diabetes mellitus, vaccine side effects, vaccine side, vaccine, diabetes mellitus, transient hyperglycemia, covid-19 mrna vaccine

## Abstract

The development of new vaccines against the SARS-CoV-2 virus in response to the COVID-19 pandemic represents a milestone in the history of public health. However, due to the rapid development and short duration of these new vaccines, the full spectrum of side effects is not yet known. A 76-year-old man presented to the clinic for follow-up after being discharged from the emergency department for hyperglycemia. His medical history included well-controlled type 2 diabetes for two years, hypertension, and hyperlipidemia. He had recently noticed high home blood glucose readings over 400 mg/dL, and his hemoglobin A1c (mean 90-day glucose level) had increased from 6.5% to 12.6%. Notably, the patient reported having excellent health behaviors, including daily exercise, a closely monitored healthy diet, and regular blood glucose testing. After extensive endocrinology workup, the rapid change in blood glucose was thought to be due to his having recently received the COVID-19 messenger RNA (mRNA) vaccine. He was started on long- and short-acting insulin and a glucagon-like peptide-1 agonist (novel injectable type 2 diabetes medication), with improvement in blood glucose. He was tapered off all medications and remains on metformin 1,000 mg twice daily after one year.Whether the new COVID-19 mRNA vaccines directly incur hyperglycemia within certain groups of patients with diabetes is not known; thus, studies exploring the relationship between vaccine antigen binding and pancreatic function are needed.

## Introduction

The rapid development of vaccines against the SARS-CoV-2 virus in response to the COVID-19 pandemic represents a milestone in the history of public health [1]. Not only were several effective, safe COVID-19 vaccines developed and tested for safety and efficacy within a short amount of time but also several vaccines implemented completely novel delivery strategies, such as messenger RNA (mRNA)-lipid nanoparticle and adenovirus platforms, to stimulate in vivo antigen generation [2]. Traditional vaccines introduce laboratory-produced pathogen components (protein antigens) or weakened or inactivated whole pathogens (bacteria or virus) into the body to trigger an immune response; however, the new mRNA-based vaccines introduce mRNA molecules directly into cells, which provide instructions for the cells to synthesize harmless pathogen components that can trigger a specific immune response. However, importantly, some side effects and adverse events after any type of vaccination may occur within certain individuals and populations, and because the new mRNA-based vaccines have been in wide use for only a short time, the full spectrum of contraindications and specific risks associated with these vaccines are not completely known. Therefore, patients with underlying health conditions may benefit from close monitoring after vaccination with mRNA-based vaccines to identify and mitigate possible side effects.

Type 1 and type 2 diabetes are chronic metabolic conditions characterized by the body either not producing or not responding to the molecule insulin, which results in a lack of blood glucose (sugar) control leading to abnormally high blood glucose levels, a situation that can be serious or even deadly. While type 1 diabetes must be treated with insulin replacement, type 2 diabetes can be treated with various oral and injectable medications and lifestyle modifications. Notably, individuals with type 2 diabetes, which can develop in adulthood, are at a higher risk of serious illness and mortality from COVID-19 [3-6], and having type 2 diabetes may also be associated with post-acute sequelae of COVID-19, or “long COVID” [7]. This heightened risk from COVID-19 suggests that vaccination should be particularly prioritized for individuals with type 2 diabetes. However, SARS-CoV-2 vaccinations may also pose specific risks for those with type 2 diabetes, although the range and severity of vaccine-related adverse events in this patient population are not clear [8]. Several cases of vaccine-related hyperglycemia have been reported [9], and poor glycemic control in patients with type 2 diabetes was shown to be associated with a low post-vaccination immune response and an increased incidence of breakthrough infections [10]. Therefore, gathering data on occurrences of hyperglycemia and other types of metabolic dysfunction following COVID-19 vaccination in individuals with type 2 diabetes will be important for determining what patient-specific and vaccine-specific factors may be associated with loss of glycemic control after vaccination. We present the case of a 76-year-old man with type 2 diabetes who experienced a bout of hyperglycemia two months after receiving a SARS-CoV-2 mRNA vaccine in the absence of other possible contributing factors. This case highlights the possibility that transient hyperglycemia may be seen after COVID-19 mRNA vaccination even in patients with well-controlled blood glucose and excellent health habits, underlining the importance of close glucose monitoring in patients with type 2 diabetes after vaccinations.

## Case presentation

A 76-year-old man with type 2 diabetes, hypertension, and hyperlipidemia presented to the family medicine clinic for follow-up care after having been discharged from the emergency department two months previously for an acute case of hyperglycemia. The patient went to the emergency department because his home glucose testing indicated blood glucose levels at approximately 400 mg/dL (healthy range <140 mg/dL). In the emergency department, he had been given intravenous fluids, and when his blood glucose levels improved, he was advised to seek a follow-up evaluation with his primary care provider.

At the time of follow-up, the patient had a normal body mass index of 23.5 kg/m^2^ and was taking daily nifedipine (90 mg) and losartan hydrochlorothiazide (50-12.5 mg) for hypertension, daily atorvastatin (40 mg) for hyperlipidemia, and metformin (a drug that lowers liver glucose production and reduces glucose uptake in the gut) 1,000 mg twice daily for type 2 diabetes, which he had been taking for approximately seven years. He was diagnosed with type 2 diabetes in 2014 and was initially placed on metformin and insulin. He took insulin for a very short time and was transitioned to glipizide (a drug that increases insulin production in the pancreas). His last time taking glipizide was in 2015, and he had been taking metformin since. He reported having been an Olympic long-distance runner and that he exercised daily (running, rowing, and weight-lifting), ate a healthy and closely monitored diet, and checked his blood glucose every day. Notably, the patient had received two doses of the Moderna COVID-19 mRNA vaccine. His first dose was received on January 27, 2021, and his second dose was received on February 24, 2021, which was two months before his emergency department presentation on April 20, 2021. Antibody titers were not measured after vaccination, so the strength of the immune response had not been assessed. Importantly, the patient reported that his blood glucose levels were normal at 100-140 mg/dL before having received the vaccinations and that the elevation in glucose started a few days after having received the second Moderna COVID-19 mRNA vaccine. Over the previous year, he had also lost approximately 8 kg of weight for unexplained reasons.

At the follow-up meeting in the family medicine clinic, the patient reported no recent changes in medication use, no changes in diet or exercise, no glucocorticoid use, and he said that he had never tested positive for COVID-19. Laboratory testing revealed elevated hemoglobin A1c at 12.6% (a measure of the mean 90-day glucose level), which was increased from 6.5% as tested approximately 15 months and 22 months (February 2020 and August 2019) previously (healthy range <5.7%). Because of this severe elevation in hemoglobin A1c, VIPoma or pancreatic mass was suspected. However, computed tomography imaging of the pancreas did not reveal any pancreatic mass, and computed tomography of the pelvis did not show a pelvic mass or infection. Because of the rise in hemoglobin A1c and hyperglycemia, the patient was prescribed dulaglutide 0.75 mg/mL weekly, which is a novel glucagon-like protein-1 (GLP-1) receptor agonist injection for treating type 2 diabetes (enhances insulin production in the pancreas and lowers glucose production in the liver).

Seven days later, the patient was admitted to an endocrinology clinic and evaluated for the possibility of type 1 diabetes. Glutamic acid decarboxylase antibodies were <5 IU/mL (reference range = <5 IU/mL), islet cell antibody test was normal at <1:4, C-peptide was 0.9 ng/mL (reference range = 0.8-4.0 ng/mL), anti-zinc transporter 8 antibody was within the healthy range at <10 U/mL, and glucose was elevated at 398 mg/dL (reference range = <99 mg/dL). Thus, laboratory results did not indicate type 1 diabetes. The patient was then started on insulin glargine (15 U daily) and insulin aspart (5 U with meals) injections. Additionally, the patient was given a Dexcom continuous glucose monitor (Dexcom, Inc., San Diego, CA). The patient then went on to experience hypoglycemia with this regimen after 21 days. Therefore, preprandial insulin aspart was stopped, insulin glargine was decreased to 10 units daily, and dulaglutide was increased to 1.5 mg/mL weekly. After 21 days, the patient’s hemoglobin A1c decreased to 10.5%, and five months after that to 6.2%, following which he was discharged from the endocrinology clinic.

The patient was seen for a follow-up visit four months after discharge from the endocrinology clinic and reported having more energy and having gained back about 6 kg of weight. He reported continued regular exercise and consistent close monitoring of his blood glucose levels, which remained stable. Because no clear causes of the patient’s hyperglycemia were found, including a lack of evidence for developing type 1 diabetes, physicians felt that there was a high likelihood that the patient’s lack of glycemic control may have been secondary to COVID-19 mRNA vaccination. The patient was eventually tapered off all glycemic medications except for metformin, and he was advised that he could receive a Moderna vaccine booster with close blood glucose monitoring; however, the patient was hesitant and stated that he would think about it.

## Discussion

In this case, emergent hyperglycemia in a previously healthy patient who had well-controlled type 2 diabetes and excellent health habits was seen approximately two months after receiving a second dose of the Moderna COVID-19 mRNA vaccine. While imaging analysis and laboratory tests ruled out pancreatic mass and developing type 1 diabetes, and because the patient’s blood glucose and hemoglobin A1c normalized after correction of hyperglycemia, the possibility exists that the patient’s unusual lack of glycemic control may have been triggered by vaccination.

Several cases of hyperglycemia in patients with preexisting diabetes within the setting of COVID-19 vaccination have been reported. One case series described three patients with type 2 diabetes who developed hyperglycemia and/or diabetic ketoacidosis about 2-10 days after receiving a COVID-19 mRNA vaccine (one Pfizer first dose and two Moderna first doses) [11]. As with our patient, temporary insulin therapy led to hyperglycemia resolution in all three patients within a short time, and notably, two of the patients had a greater than 5% increase in hemoglobin A1c, similar to our patient. Another series described transient hyperglycemia in three patients with type 2 diabetes who had received the Indian Covishield (ChAdOx1 nCoV-19) vaccine, which unlike the Moderna and Pfizer vaccines, uses the adenovirus vector developed by AstraZeneca [12]. Blood glucose returned to normal within 3-15 days with no interventions in two patients, while a third patient received an increase in metformin therapy and had blood glucose normalized within one month [12]. As with our patient, all patients described by Mishra et al. had reported good adherence to medication, exercise, and a healthy diet. Another series reported three cases of acute hyperglycemia in three patients that occurred 20-36 days after a first dose of the AstraZeneca ChAdOx1 nCoV-19 adenoviral vaccine [13]. In this series, two of the three male patients had pre-diabetes and all had additional comorbidities, including dyslipidemia and obesity; notably, hemoglobin A1c was elevated for all three men, although the timing of this increase could not be determined [13].

Furthermore, cases of new-onset diabetes and hyperglycemia have been reported after COVID-19 vaccination. For instance, one man presented with severe hyperglycemia (>1,200 mg/dL) within one month of receiving two doses of the Pfizer-BioNTech COVID-19 vaccine, and as with our patient, he had underlying hypertension [14]. In another case from Japan, a woman with no history of metabolic dysfunction presented with diabetic ketoacidosis and hyperglycemia within two weeks of receiving the Pfizer-BioNTech vaccine [15]. Genetic analysis of this patient revealed that she had a human leukocyte antigen haplotype that is associated with type 1 diabetes in Japan [15]. Collectively, these rare occurrences of metabolic dysfunction after COVID-19 vaccination suggest that underlying causes, or perhaps a combination of underlying causes, may make some individuals more susceptible to transient, treatable loss of glycemic control after vaccination.

Most cases involving loss of glycemic control after COVID-19 vaccination occurred relatively shortly after vaccine administration, whereas our patient’s hyperglycemia occurred 1-2 months after his second vaccination. However, we note that our patient was emphatic about his adherence to diet, exercise, and monitoring of his blood glucose and that other factors that could have contributed to our patient’s acute hyperglycemia, such as unreported medications, recent stressful events, or transient infections, were likewise ruled out. Indeed, we note that his unexplained weight loss that developed over one year before vaccination, which was remediated after treatment, is curious and suggests that some unknown confounding factor may have played a role in this patient’s trajectory. Our patient was also at a rather advanced age, 76 years, and this could have contributed to a delayed post-vaccination immune response and the metabolic derangement; however, because antibody titers were not measured after vaccination, we cannot know the rate at which his immune system responded to the vaccine. Overall, longitudinal studies assessing multiple factors after vaccination in adults at advanced age might shed light on the mechanism of vaccination in a vulnerable population.

Why patients might experience a loss of glycemic control after COVID-19 vaccination is not known. However, the SARS-CoV-2 virus binding protein, angiotensin-converting enzyme 2, is expressed in the human pancreas [16], and exploration of whether vaccine antigen binding to angiotensin-converting enzyme two in the pancreas might instigate inflammation or changes in glucose metabolism would be informative. We note that the anecdotal evidence for COVID-19 vaccination causing hyperglycemia in individuals with diabetes has been compelling enough to inspire at least one recently completed large retrospective study (NCT04923386) and one prospective cohort study that is currently recruiting subjects (NCT05233592), and the results from these controlled clinical studies will hopefully shed light on this phenomenon.

## Conclusions

Overall, whether novel COVID-19 vaccines directly incur hyperglycemia within certain groups of patients with diabetes is not known. Thus, studies exploring the relationship between vaccine antigen binding and pancreatic function are needed. Because glucose control and underlying diabetes are so strongly associated with poor outcomes from COVID-19, close blood glucose and metabolic monitoring of patients with type 1 or type 2 diabetes after vaccination is warranted.
